# Pilot testing of a dementia literacy intervention for Korean American elders with dementia and their caregivers

**Published:** 2021-10-30

**Authors:** Hae-Ra Han, Scott Choi, Jinhee Wang, Hochang Ben Lee

**Affiliations:** ^1^The Johns Hopkins University, School of Nursing, Baltimore, MD, United States; ^2^The Johns Hopkins University, Bloomberg School of Public Health, Baltimore, MD, United States; ^3^Department of Nursing, Inha University, Incheon, Republic of Korea; ^4^Korean Community Service Center of Greater Washington, Annandale, VA, United States; ^5^University of Rochester, School of Medicine, Rochester, NY, United States

**Keywords:** dementia, early diagnosis, literacy, Korean American

## Abstract

**Background and Aim:**

To test the feasibility and acceptability of a dementia literacy intervention for Korean American (KA) elders with dementia and their caregivers: K-PLAN (**P**reparing successful aging through dementia **L**iteracy education **A**nd **N**avigation).

**Method:**

Twenty cognitively impaired Korean elders (Clinical Dementia Rating 1+) and their caregivers participated in a pilot trial to receive the K-PLAN intervention, which consisted of 1-h dementia literacy education followed by monthly phone counseling sessions and navigation assistance for 3 months by a trained bilingual community health worker. Outcomes of interest were linkage to medical services for dementia evaluation (KA elders) and dementia literacy, social support, self-efficacy in dementia care, depression, and quality of life (caregivers). Using a one-group pre-and post-test design, all ten dyads were followed up at 12 weeks.

**Results:**

The K-PLAN intervention was highly feasible and acceptable. We were able to retain all twenty participants over the study period (100% retention rate). In addition, 100% of the caregivers would recommend the program with an overall satisfaction rating of 9.7 on a 1–10-point scale. Three of the elders (30%) were linked to medical services for dementia by medical record review. The effect sizes for caregiver outcomes ranged from 0.4 to 0.7 in absolute value.

**Conclusion:**

Dementia literacy intervention has the potential to promote linkage to medical services for dementia evaluation and early diagnosis among linguistically isolated KA elders while improving caregiver psychological outcomes. Studies with larger sample sizes, comparison groups, and cost-effective analyses are needed to inform the application of K-PLAN in diverse community settings.

**Relevance for Patients:**

Early diagnosis of dementia can help preserve functional status. Promoting dementia literacy and linkage to health services through community-based programs such as K-PLAN may enable underserved racial/ethnic minority communities to make timely follow-up for dementia evaluation and care planning.

## 1. Introduction

Despite their higher prevalence of dementia, ethnic minority elders in the United States are underdiagnosed and at greater risk of not receiving appropriate care than their white counterparts [1,2]. This trend applies to Korean Americans (KAs), who have a higher prevalence of dementia compared to than the general elderly population in the U.S. (20% vs. 6 – 13%) [3,4].

Promoting linkage to medical services for dementia has significant benefits because early diagnosis and follow-up preserves functional status [3]. However, many caregivers are often left to navigate the referral processes for probable dementia before necessary diagnostic evaluation can be performed, resulting in confusion, stress, and anxiety [5]. This is particularly so for KA families due to substantial language barriers, limited health literacy, lack of self-confidence and social support, and lack of bicultural and bilingual mental health providers [4].

Using a community-engaged approach, we developed a dementia literacy-enhanced intervention program for KA elders with probably dementia—K-PLAN (**P**reparing successful aging through dementia **L**iteracy education **A**nd **N**avigation). We hypothesized that participation in the intervention would be associated with improved linkage to medical services for dementia (i.e., formal evaluation by a medical provider) and improvements in psychosocial variables among caregivers.

## 2. Materials and Methods

### 2.1. Design and sample

We used a single-arm pre-and post-test design for this study and assessed the feasibility, acceptability, and preliminary efficacy of the K-PLAN among KA elders with dementia and their caregivers. Community-dwelling KA elders were recruited by Mini-Mental State Exam screening in the community, word-of-mouth, advertisements in ethnic newspapers, and referrals from a free community clinic for uninsured patients. Eligibility criteria for KA elders (65+ years) were: (1) Residence in Baltimore-Washington Metropolitan Area, (2) Clinical Dementia Rating 1+, and (3) no prior formal diagnosis of dementia by a healthcare provider. Eligibility criteria for caregivers (18+ years) were: (1) residence in the Baltimore-Washington Metropolitan Area, and (2) had at least weekly contact with a KA elder with probable dementia. Ten eligible dyads completed the study assessment at baseline and again at 12 weeks (100% retention).

### 2.2. K-PLAN intervention

The study intervention, led by a trained bilingual community health worker (CHW), consisted of dementia literacy education followed by monthly phone counseling over 12 weeks. CHWs are frontline paraprofessionals who have a close understanding of the community they serve [6]. For the purpose of this study, two bilingual CHWs were identified. The CHWs were community staff members at a local community organization with more than 30 years of service history in the target community. Both CHWs received a total of 12 h of training on the K-PLAN intervention by the study team. The training included detailed instructions about how to conduct each component of study interventions. One of the CHWs moved out of state before the intervention began; hence, the intervention was delivered by one CHW.

The K-PLAN is theory-driven and builds on von Wagner’s health literacy model [7] to incorporate key elements such as dementia literacy, dementia knowledge, and self-efficacy for better patient outcomes. The K-PLAN was developed in partnership with a community advisory committee (e.g., caregivers of KA elders with dementia, a CHW, clinicians, and bilingual researchers) to explore preferences for desired intervention materials, approaches, and outcomes. Using a picture guidebook, a trained bilingual CHW delivered dementia literacy education to cover causes and diagnosis of dementia, steps taken to receive a dementia screening test by a healthcare provider, working with a primary care provider and a geriatric specialist to plan for dementia care after the diagnosis. After completion of the educational session, the CHW used a structured counseling form to provide phone counseling. The main goals of monthly counseling were: (1) To assess the caregiver’s self-efficacy obtaining medical services for dementia evaluation; and (2) to work with the dyad on strategies to manage identified barriers and provide individually tailored support and referrals for navigation assistance.

### 2.3. Procedures

The Johns Hopkins Medicine Institutional Review Board approved the study protocol. Upon completion of the baseline assessment, the CHW delivered 60 – 90-min dementia literacy education sessions within 1 – 2 weeks and monthly thereafter for 12 weeks. Sessions were conducted face-to-face at the participant’s home or at the office of a community partner organization. At each follow-up phone counseling session, the CHW checked and discussed the caregiver’s progress toward arranging a formal evaluation of dementia by a healthcare provider by identifying a primary care or memory clinic, or by making an appointment for an evaluation. The CHW answered relevant questions and provided necessary navigational assistance; for instance, identifying a place for the patient to receive a formal dementia evaluation where bilingual services were available, enrolling the patient in Medicare or Medicaid to minimize cost barriers in preparation for a clinic visit, making a clinic appointment; or referral to an adult daycare center. A trained bilingual research assistant collected data at baseline and 12 weeks from the start of the intervention by face-to-face interviews at a community location. In addition, caregivers who completed the study were invited to participate in a post-intervention interview to share their experiences. Fifty dollars were provided to each participating dyad at each data collection visit and to caregiver participants who joined the post-intervention interview.

### 2.4. Measurement

The sociodemographic and medical characteristics of the participants were assessed at baseline using a questionnaire developed for the purposes of this study. Study variables were measured at baseline and 12 weeks from the start of the intervention. Data were collected regarding these outcomes: linkage to medical services for dementia (primary or specialty care evaluation for cognitive impairment), caregiver psychosocial variables (dementia literacy, social support, self-efficacy, and depression), and caregiver quality of life.

Upon participant’s pre-approval, linkage to medical services for dementia evaluation was verified by a medical records review. Korean translated versions of each tool were validated and reliable. Caregiver psychosocial outcomes were measured by the following translated, validated study instruments: (a) 10-item dementia literacy test with one point was assigned for each correct response; higher scores reflected higher dementia literacy [2]; (b) 8-item modified Medical Outcomes Study—Social Support survey [8], which assess participant’s perceptions of support availability via a Likert scale; total scores ranged from 8 to 40, with higher scores indicating higher social support; (c) the Dementia Self-Efficacy Scale consists of 10 items with Likert scales measuring caregiver confidence in in handling problems (higher scores indicated higher dementia self-efficacy) such as memory loss, frustrations of caring or finding organizations or agencies that provide services for dementia care [9]; (d) severity of depressive symptoms based on the Diagnostic and Statistical Manual of Mental Disorders, 4^th^ edition – the Patient Health Questionnaire-9 [10]; a total range of 0 – 27 with cut-offs at 5, between 5 – 9, and 10+ (indicating minimal depression, mild-moderate, or higher levels of depression, respectively); and (e) caregiver quality of life: Quality of Life–Dementia Caregiver Scale – participants provided ratings along thirteen domains (physical health, energy, mood, living situation, memory, family, family, marriage, friends, self as a whole, ability to do chores, ability to do things for fun, money, and life as a whole; rating each on a scale of 1 – 4), with higher scores indicating better quality of life [11].

Finally, we collected data on study recruitment and retention, attendance at dementia literacy education sessions, and success rates of phone counseling. Acceptability was measured using a 0-10 Likert scale regarding satisfaction. Other information was retrieved from our intervention delivery logs and weekly research team reports.

### 2.5. Analysis

We performed the analyses using data from the 20 KA older adults with dementia and their caregiver participants who completed all data points. We used descriptive statistics to summarize sample characteristics and study variables. The primary outcome was linkage to medical services for dementia (i.e., having had primary care or specialty care provider evaluation for cognitive impairment). Effect sizes were calculated using the mean change from baseline to 12-weeks follow-up divided by the baseline standard deviation [12].

## 3. Results

### 3.1. Sample characteristics

The KA elderly sample comprised mostly females (90%) and older (mean age = 81.5 years) with a low level of education (less than high school = 60%). The majority were insured (80%) and had a Korean primary care physician (90%). The KA caregiver sample comprised half females (50%) with a mean age of 61 years, had a high school diploma (90%) or above, and were employed full- or part-time (60%). The caregivers predominantly rated themselves as having limited English proficiency (90%). As for each caregiver’s relationship to a patient, 50% were adult children or children-in-law, 40% were spouses, and 10% were church friends (Table 1).

**Table 1:** Sample characteristics at baseline (n = 20)

Variable	Median (IQR)^†^or %
KA older adults (n = 10)	
Age, years	81.5 (78.2 – 86.2)
Female	90.0
<High school	60.0
Insured	80.0
Have Korean PCP	90.0
CDR	1.0 (1.0 – 2.0)
Caregivers (n=10)	
Age, years	61.0 (56.0 – 82.7)
Female	50.0
<High school	10.0
Employed	60.0
Difficulty with English	90.0
Relationship to patient	
Child/child-in-law	50.0
Spouse	40.0
Church friend	10.0

† Inter-quartile range (IQR=75^th^ percentile minus 25^th^ percentile)

### 3.2. Outcome changes

Table 2 compares the study outcomes at baseline and 12 weeks. Three of the elders (30%) received formal dementia assessment by a specialist or primary care physician at 12 weeks, as confirmed by a medical records review. The effect sizes for caregiver outcomes ranged from 0.4 to 0.7 in absolute value. At 12 weeks, the mean changes in the caregiver outcomes were all in expected directions. Specifically, dementia literacy, social support, and dementia self-efficacy increased by 1.0 (SD = 2.4), 7.3 (9.6), and 13.7 (SD = 23.6), respectively, with effect sizes ranging from 0.4 to 0.7. Depression and quality of life also improved at 12 weeks, with an absolute effect size of 0.4 for both.

**Table 2:** Changes in caregiver outcomes over 12 weeks

Variable	Median (IQR)		Mean change (SD)	Effect size^†^

	Baseline	12 weeks		
Dementia literacy	4 (1 – 10)	6 (2 – 10)	1.0 (2.4)	0.4
Social support	26 (18 – 34)	38 (27 – 40)	7.3 (9.6)	0.7
Self-efficacy	43 (18 – 78)	65 (30 – 85)	13.7 (23.6)	0.5
Depression	6 (0 – 11)	6 (0 – 6)	−1.2 (3.0)	−0.4
Quality of life	27 (22 – 35)	30 (25 – 39)	2.9 (6.4)	0.4

† Mean change from baseline to 12 weeks divided by the standard deviation of the mean at baseline

### 3.3. Feasibility and acceptability

We achieved a retention rate of 100% at 12 weeks. Treatment delivery and receipt was highly consistent. All participants received full components of the intervention. Every caregiver participant received dementia literacy education and completed on average about 3 (mean = 2.6) phone counseling sessions (range = 1–3). This study also demonstrated good treatment enactment (whether participants sought to obtain a dementia diagnosis), and 30% of the caregivers had their elders receive a formal dementia diagnosis by the end of the study period (12 weeks).

The K-PLAN intervention received an overall satisfaction rating of 9.7 on a 10-point scale, and 100% of the caregiver sample reported that they would recommend the program to other caregivers. Post-intervention interviews revealed improved understanding of dementia, for instance, a daughter-in-law caregiver said:

 This program made me realize that [my mother-in-law] was showing signs of dementia and I began to accept that this may be a new normal for her.

Participants also appreciated the ongoing support provided by the bilingual CHW and pointed out that the CHW support completion of formal dementia evaluation. A husband who had taken care of his wife noted:

 I think phone counseling was the best thing that this program offered… I wouldn’t have taken my wife to the clinic if it wasn’t for [the CHW’s] call… The phone check-ins really kept me on track.

Finally, there were several comments addressing the usefulness of CHW navigation to address access barriers. A caregiver stated:

 I sensed that [the care recipient] might have had dementia for some time. I wanted her to see a doctor, but she couldn’t because of her insurance status… Then I learned out about this program and found it beneficial.

## 4. Discussion

To the best of our knowledge, the current study was the first to focus on methods to actually address dementia literacy, which has been shown to be consistently low among ethnic minority elders [13,14]. Findings from this pilot trial indicate that the K-PLAN yielded promising outcomes. Our focus on dementia literacy education and follow-up support was feasible and highly acceptable. Post-intervention evaluations revealed high satisfaction ratings and consistently positive comments about our bilingual CHW by post-intervention interviews with the caregiver participants. These results demonstrate the trust, knowledge and understanding about the topic, genuine attention and care, and flexibility that the trained bilingual CHW was able to deliver. In particular, the CHW represented a local community organization with more than three decades of service in the target community. We believe such long-term social service rendered by the community organization and the CHW working within the organization might have contributed to our success including the retention rate of 100%. Nevertheless, the short-term follow-up period of 12 weeks did not allow the study team to capture the full impact of some of the navigational assistance (e.g., processing Medicare) and intervention on linkage to care; a longer-term follow-up with a larger sample is warranted.

There are study limitations. As there was no control group, and the study sample was a small convenience sample, the generalizability of the study findings is limited. Nevertheless, the effect sizes estimated for the study variables were highly encouraging. Another limitation is that the sample included KAs only. This targeted approach was due to the profound disparities in dementia care reported among KAs [2,4].

In conclusion, our results will aid clinicians and researchers in understanding the essential attributes of dementia care transition needed for elders with undiagnosed dementia. Future interventions should provide dementia literacy education and navigation support to enable families of KA elders with dementia to adequately plan for the potential challenges of caregiving.
